# Cathelicidin Antimicrobial Peptide Acts as a Tumor Suppressor in Hepatocellular Carcinoma

**DOI:** 10.3390/ijms242115652

**Published:** 2023-10-27

**Authors:** Lien-Hung Huang, Cheng-Shyuan Rau, Yueh-Wei Liu, Hui-Ping Lin, Yi-Chan Wu, Chia-Wen Tsai, Peng-Chen Chien, Chia-Jung Wu, Chun-Ying Huang, Ting-Min Hsieh, Ching-Hua Hsieh

**Affiliations:** 1 Department of Neurosurgery, Kaohsiung Chang Gung Memorial Hospital and Chang Gung University College of Medicine, Kaohsiung 833, Taiwan; ahonbob@gmail.com (L.-H.H.); ersh2127@adm.cgmh.org.tw (C.-S.R.); 2 Department of General Surgery, Kaohsiung Chang Gung Memorial Hospital and Chang Gung University College of Medicine, Kaohsiung 833, Taiwan; anthony0612@adm.cgmh.org.tw; 3 Department of Trauma Surgery, Kaohsiung Chang Gung Memorial Hospital and Chang Gung University College of Medicine, Kaohsiung 833, Taiwan; poppy952@gmail.com (H.-P.L.); janewu0922@gmail.com (Y.-C.W.); flying011401@gmail.com (C.-W.T.); venu_chien@hotmail.com (P.-C.C.); alice8818@yahoo.com.tw (C.-J.W.); junyinhaung@yahoo.com.tw (C.-Y.H.); hs168hs168@gmail.com (T.-M.H.); 4 Department of Plastic Surgery, Kaohsiung Chang Gung Memorial Hospital and Chang Gung University College of Medicine, Kaohsiung 833, Taiwan

**Keywords:** HCC (hepatocellular carcinoma), iTRAQ (isobaric tags for relative or absolute quantitation), CAMP (cathelicidin antimicrobial peptide)

## Abstract

Hepatocellular carcinoma (HCC) is associated with high rates of metastasis and recurrence, and is one of the most common causes of cancer-associated death worldwide. This study examined the protein changes within circulating exosomes in patients with HCC against those in healthy people using isobaric tags for a relative or absolute quantitation (iTRAQ)-based quantitative proteomics analysis. The protein levels of von Willebrand factor (VWF), cathelicidin antimicrobial peptide (CAMP), and proteasome subunit beta type-2 (PSMB2) were altered in HCC. The increased levels of VWF and PSMB2 but decreased CAMP levels in the serum of patients with HCC were validated by enzyme-linked immunosorbent assays. The level of CAMP (the only cathelicidin found in humans) also decreased in the circulating exosomes and buffy coat of the HCC patients. The serum with reduced levels of CAMP protein in the HCC patients increased the cell proliferation of Huh-7 cells; this effect was reduced following the addition of CAMP protein. The depletion of CAMP proteins in the serum of healthy people enhances the cell proliferation of Huh-7 cells. In addition, supplementation with synthetic CAMP reduces cell proliferation in a dose-dependent manner and significantly delays G1-S transition in Huh-7 cells. This implies that CAMP may act as a tumor suppressor in HCC.

## 1. Introduction

Hepatocellular carcinoma (HCC) is the majority type of primary liver cancer and is one of the most common causes of cancer-associated death worldwide [1]. The risk factors of HCC occurrence include cirrhosis, an infection with hepatitis B or C virus, heavy alcohol consumption, and non-alcoholic fatty liver disease [2]. The average 5-year survival rate is only approximately 20%, despite significant advances in diagnostic and therapeutic strategies for HCC [3]. High rates of metastasis and recurrence account for poor survival in HCC. The 1-year risk of recurrence after surgery is ≥10% and reaches 70–80% after 5 years [4].

Hence, it is imperative to elucidate the mechanisms driving the progression of HCC and to pinpoint the risk factors or develop potential biomarkers for improving its early diagnosis, prognostic assessment, and therapeutic targeting.

Exosomes are low-density (1.13–1.19 g/mL), small-sized (30–150 nm) extracellular vehicles with a lipid bilayer membrane [5]. Exosomes can be found in various body fluids, since almost all eukaryotic and prokaryotic cell types secrete exosomes [6]. Exosomes carry a complex cargo of proteins, nucleic acids, and lipids, and their composition is highly dependent on the cell type of origin and its state. They mediate cell-to-cell communication and signaling through their functional cargo [7]. In tumor development, exosomes are involved in crosstalk between tumor cells and their surrounding microenvironment, which facilitates tumor development and progression, influences the immune system, or even drug resistance [8]. In HCC, cancer-cell-derived exosomes contain RNAs that affect the epithelial–mesenchymal transition, surrounding cell proliferation, migration, and drug resistance [9,10,11]. These uniquely expressed protein or RNA profiles also reflect the characteristics of tumor cells.

This study examined the protein changes within circulating exosomes in patients with HCC compared to healthy subjects using isobaric tags for a relative or absolute quantitation (iTRAQ)-based quantitative proteomics analysis. The protein levels of von Willebrand factor (VWF), cathelicidin antimicrobial peptide (CAMP), and proteasome subunit beta type-2 (PSMB2) were altered in HCC. CAMP (also known hCAP-18 or FALL-19) is the only cathelicidin found in humans, and is known for its antibacterial and immunomodulatory activities [12]. The topology of CAMP contains an N-terminal signal peptide, cathelin domain, and C-terminal antimicrobial peptide region (LL-37) [13]. CAMP is a proprotein that is stored in granules and lamellar bodies. The mature antimicrobial peptide (LL-37) is released as a functional peptide after enzymatic cleavage, with moderate antibacterial and antibiofilm activities [14]. This study found that CAMP is present in circulating exosomes in a full-length or N-terminally cleaved form. The CAMP protein level decreased in the serum and circulating exosomes of patients with HCC. A high CAMP concentration delayed the G1-S transition and decreased the cell proliferation of Huh-7 cells. In addition, CAMP protein depletion in the serum of healthy subjects can enhance the cell proliferation of Huh-7 cells. Our data suggest that CAMP plays an important role in inhibiting tumor development during HCC development.

## 2. Results

### 2.1. Characterization of Proteins within Circulating Exosomes

We examined the differentially expressed proteins in the circulating exosomes in HCC using an iTRAQ-based quantitative proteomics analysis. Circulating exosome proteins were labeled with 4-plex iTRAQ reagents of different weights. We identified 420 exosomal proteins and quantified 347 circulating exosomal proteins through a liquid chromatography tandem mass spectrometry (LC-MS/MS) analysis. A list of the identified exosomal proteins is summarized in Supplemental Table S1. The protein levels of 11 exosomal proteins were different in the patients with HCC compared to the healthy controls (Table 1). Five of these eleven exosomal proteins were up-regulated in patients with HCC, including histone H4 (HIST1H4A), apolipoprotein C-II (APOC2), CAMP, PSMB2, and VWF. Six of the eleven were down-regulated in patients with HCC, including matrix metalloproteinase-9 (MMP9), insulin-like growth factor-binding protein complex acid labile subunit (IGFALS), immunoglobulin lambda-like polypeptide 5 (IGLL5), sex hormone-binding globulin (SHBG), insulin-like growth factor-binding protein 3 (IGFBP3), and lactotransferrin (LTF).

### 2.2. Protein Candidate Validation

**Figure 1:**
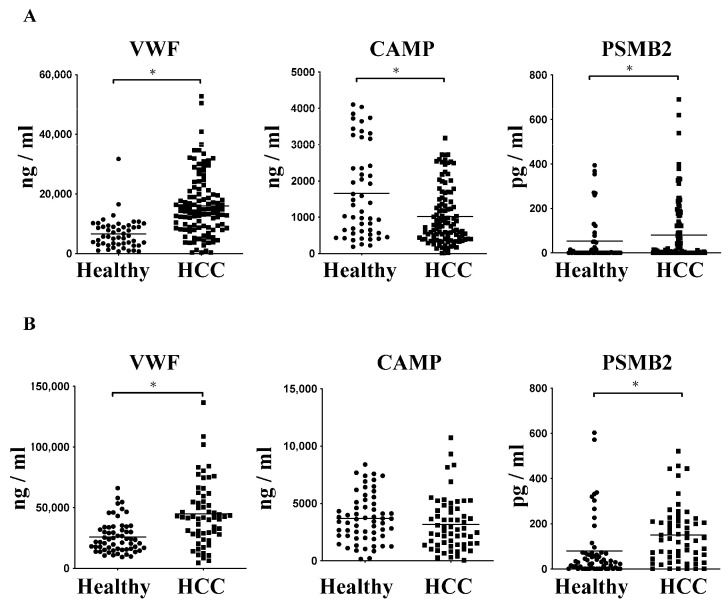
The expression of candidate proteins in HCC. The protein levels of VWF, CAMP, and PSMB2 in (**A**) serum and (**B**) circulating exosomes of HCC patients vs. those in healthy controls quantified by enzyme-linked immunosorbent assay (ELISA); * indicated a significance of *p* < 0.05.

We used enzyme-linked immunosorbent assays (ELISAs) to confirm the protein levels of the candidate proteins that were up-regulated in HCC. HIST1H4A was excluded, since it is mainly expressed in the nucleus. Initially, 64 serum samples from 43 patients with HCC and 21 healthy controls were evaluated. The protein levels of VWF and PSMB2 increased in the HCC serum (Supplemental Figure S1). The protein level of APOC2 was not different between the healthy control subjects and patients with HCC. However, the serum CAMP protein level in patients with HCC was significantly decreased. Further, omitting APOC2 for verification, the expressions of VWF, CAMP, and PSMB2 were examined in a total of 162 serum samples from 112 patients with HCC and 50 healthy controls in the tissue bank. The VWF and PSMB2 protein levels significantly increased in the HCC serum compared to that from the healthy controls (Figure 1A). The CAMP protein level was significantly decreased in the HCC serum.

Subsequently, we examined the protein levels of VWF, CAMP, and PSMB2 in a total of 120 circulating exosomes from 60 patients with HCC and 60 healthy controls. The protein levels of VWF and PSMB2 significantly increased in the HCC circulating exosomes compared to the healthy controls (Figure 1B). These results indicated that the protein levels of exosome proteins correlated with the serum protein levels, except that the CAMP protein level in HCC circulating exosomes, which were insignificantly lower.

### 2.3. The Expression Profiles of CAMP in HCC

**Figure 2:**
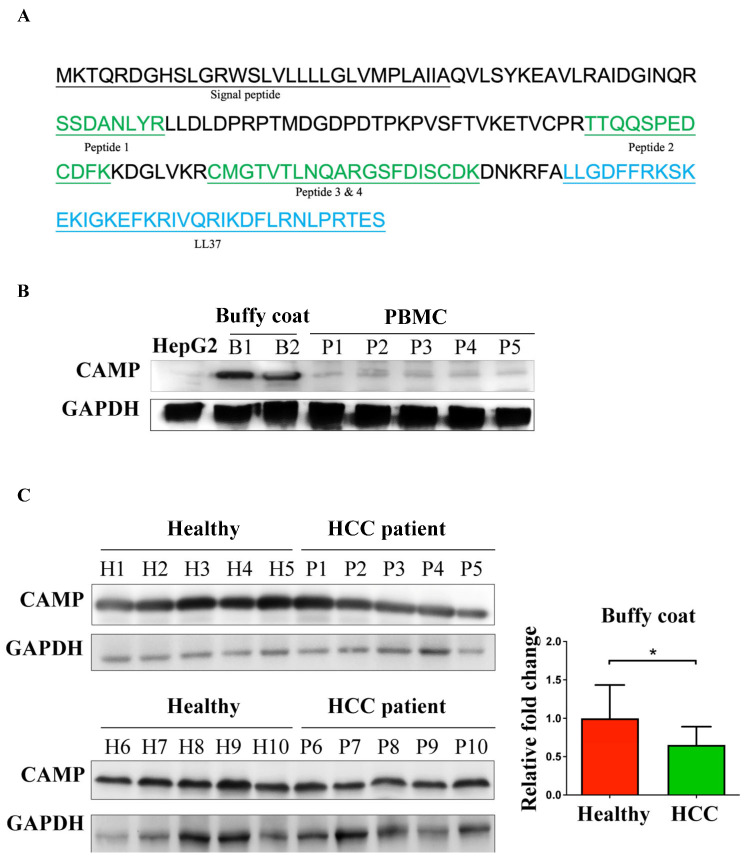
CAMP expression profile in HCC. (**A**) Schematic showing the CAMP protein sequence. The peptides identified using liquid chromatography tandem mass spectrometry (LC MS/MS) are marked in green. LL-37 peptide sequences are marked in blue. (**B**) The expression profile of CAMP in HepG2 cells, buffy coat, and peripheral blood mononuclear cells (PBMCs) were examined using Western blotting. (**C**) The CAMP protein level was significantly decreased in the buffy coat from patients with HCC compared with healthy subjects; * indicated a significance of *p* < 0.05.

Four unique peptides belonging to CAMP were identified using MS/MS data. The sequence alignment showed that CAMP is present in the serum in a full-length or N-terminally cleaved form (Figure 2A).

CAMP is mainly expressed in the bone marrow, lymphoid tissues, and blood of male tissues according to the Human Protein Atlas Database. The CAMP expression level is similar in females (Supplemental Figure S2). CAMP was expressed to a greater degree in the buffy coat compared to the peripheral blood mononuclear cells (PBMCs) (Figure 2B). The CAMP expression was low in HepG2 cells. The CAMP protein level was significantly decreased in patients with HCC compared to healthy subjects (Figure 2C).

### 2.4. The Biological Function of CAMP in HCC

**Figure 3:**
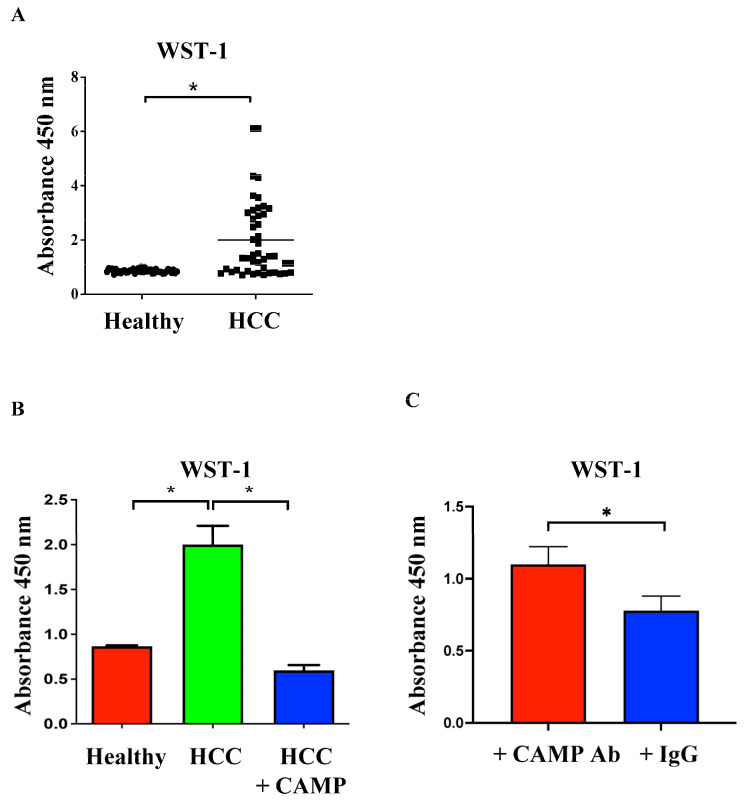
Effect of serum on HCC cell proliferation. (**A**) Huh-7 cell proliferation was stimulated by serum from HCC patients. (**B**) The proliferative effects of serum from HCC patients were reduced following the addition of CAMP proteins. (**C**) Depletion of serum CAMP using CAMP antibodies increased cell proliferation compared with those treated with IgG; * indicated a significance of *p* < 0.05.

Serum from the patients with HCC stimulated the cell proliferation of Huh-7 cells according to WST-1 assays (Figure 3A). Supplemental CAMP proteins counteracted these proliferative effects (Figure 3B). In contrast, the depletion of serum CAMP in healthy people increased cell proliferation (Figure 3C). These results indicated that a high CAMP concentration decreased cell proliferation.

**Figure 4:**
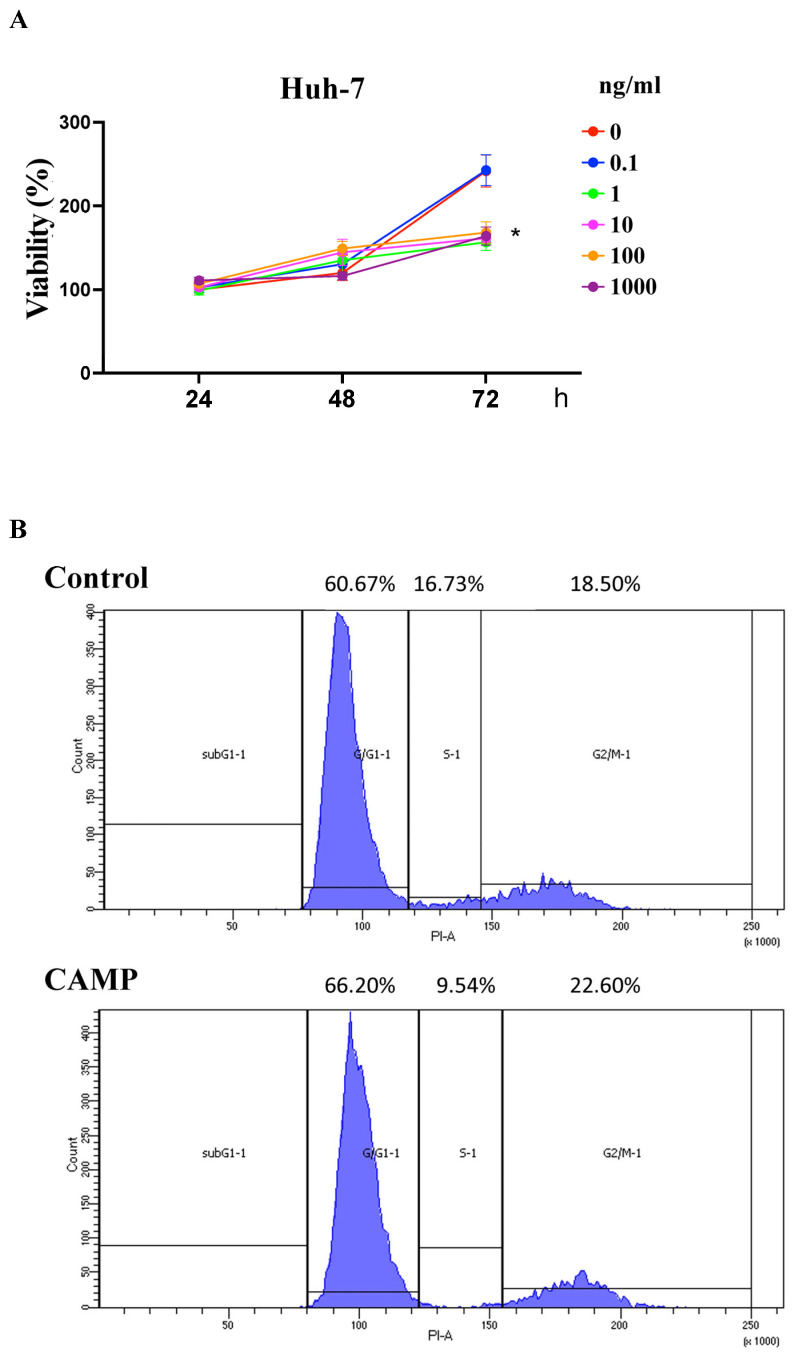
Effect of CAMP on HCC cell proliferation and cell cycle distribution. (**A**) WST-1 assays were conducted in Huh-7 cells after treatment with different CAMP concentrations for 48 h. (**B**) Cell cycle analysis was performed in Huh-7 cells after treatment with 1000 ng/mL CAMP; * indicated a significance of *p* < 0.05.

Huh-7 cell growth was diminished when the CAMP concentration was above 10 ng/mL (Figure 4A). Furthermore, supplemental CAMP significantly delayed the G1-S transition in the Huh-7 cells (Figure 4B). Together, these results indicated that CAMP acted as a tumor suppressor in the development of HCC.

## 3. Discussion

We determined that the protein levels of VWF, CAMP, and PSMB2 were altered in HCC; VWF and PSMB2 were up-regulated while CAMP was down-regulated in the HCC serum.

VWF is a multimeric glycoprotein present in blood plasma, the subendothelial matrix, and as storage granules in endothelial cells and platelets [15]. It plays a role in coagulation and hemostasis by mediating platelet adhesion and acts as a carrier molecule for procoagulant factor VIII [16]. Its levels are significantly increased in various cancers, including HCC. In addition, PSMB2 is a member of proteasome 20S core beta subunit, which plays several roles in many types of cancers, such as gastric cancer [17] or glioma [18]. Previous studies have reported that PSMB2 was regulated by NUDT21. A knockdown of NUDT21 results in an increased PSMB2 expression [19]. NUDT21 is downregulated in HCC and a low NUDT21 expression correlates with a poor prognosis in HCC patients. It can partially explain the increase in PSMB2 in HCC.

The mRNA level of LL-37 is down-regulated in HCC tissues [20]. Hepatocellular carcinoma tumor tissues have insignificantly lower levels of CAMP mRNA expression compared to those in normal liver tissues according to the Gene Expression Profiling Interactive Analysis (GEPIA) and UALCAN databases [21]. The protein levels of CAMP in HCC tissues are significantly down-regulated compared to those in adjacent normal liver tissues [21]. This study found that the CAMP protein levels significantly decreased in HCC serum and circulating exosomes. Additionally, there was a noticeable decline in CAMP protein in the buffy coat of the patients with HCC. The CAMP protein levels decreased in HCC tissue, serum, circulating exosomes, and buffy coat. This implied that the decrease in CAMP protein in serum/circulating exosomes is partly owed to the decrease in CAMP in the liver cancer tissues and buffy coat in HCC. In addition, the reduction in CAMP protein in the serum leads to increased HCC cell proliferation. This effect can be reduced by the addition of CAMP protein or enhanced by CAMP protein depletion. Synthetic CAMP reduced the cell proliferation of Huh-7 cells in a dose-dependent manner. Supplemental synthetic CAMP significantly delayed the G1-S transition in Huh-7 cells. A recent study showed that LL-37 peptide delayed G1-S transition by suppressing the CyclinD1-CDK4-p21 checkpoint signaling pathway [20]. This may be partly explained by CAMP affecting cell proliferation through cell cycle regulation.

Notedly, CAMP may either serve as a tumor promoter or a tumor suppressor in different human cancers [22,23]. CAMP displays a tumorigenic effect in ovarian cancer [24], lung cancer [25], breast cancer [26], prostate cancer [27], pancreatic cancer [28], malignant melanoma [29], and skin squamous cell carcinoma [30]. CAMP exerts anti-cancer effects in colon cancer [31], gastric cancer [32], hematologic malignancies [33], and oral squamous cell carcinoma [34]. The overexpression of CAMP through plasmid transfection promotes cell proliferation, migration, and invasion. It also enhances the endothelial–mesenchymal transition [21,35]. Although we demonstrated that the protein levels of CAMP are altered in HCC patients, cell experiments confirmed that CAMP affects cell growth and cell cycle. The molecular regulation of CAMP is unclear. Would the same result be expected in a complex organism? Many factors (including genetics, hormones, and environmental factors) can influence immune responses, and there may be subtle differences regarding CAMP regulation or function in HCC. Research on this topic is ongoing and further efforts are required to explore the role of CAMP in the development of HCC.

## 4. Materials and Methods

### 4.1. Patient Population and Clinical Specimens

This study was approved by The Chang Gung Memorial Hospital’s Institutional Review Board (IRB number, 201900911B0) and we received signed informed permission from all subjects. Circulating exosomes were extracted from 60 healthy people and 60 HCC patients. Additionally, the serum samples of 50 healthy controls and 112 patients with HCC were acquired from tissue banks at Chang Gung Memorial Hospital in Kaohsiung, Taiwan.

The iTRAQ labeling experiment used circulating exosomes from 10 healthy people (mean age: 37.6 ± 6.1 years) and 10 patients with HCC (mean age: 63.9 ± 7.1 years). The target protein within the circulating exosomes was validated using 60 healthy control subjects (mean age: 39.9 ± 9.0 years) and 60 patients with HCC (mean age: 65.4 ± 10.0 years). The target protein in the serum was validated by using 50 healthy control subjects (mean age: 34.4 ± 8.0 years) and 112 patients with HCC (mean age: 60.4 ± 11.1 years) from tissue banks. These sera were also used to verify the WST-1 assay.

### 4.2. Circulating Exosome Preparation and Exosome Protein Extraction

Five hundred microliters of serum was mixed with 126 µL of ExoQuick-TC solution (EXOTC50A-1, System Biosciences, Palo Alto, CA, USA) and refrigerated at 4 °C overnight. The supernatant was discarded after centrifugation at 1500× *g* for 30 min, and the pellet was resuspended in 50 µL of T-PER tissue protein extraction reagent (78510, Thermo Fisher Scientific, Waltham, MA, USA). The protein samples from exosomes were desalted using an Amicon^®^ Ultra-15 centrifugal unit (Merck-Millipore, Kenilworth, NJ, USA) and quantified with the bicinchoninic acid (BCA) protein assay (23225, Thermo Fisher Scientific).

### 4.3. iTRAQ-Based Quantitative Proteomic Analysis

Twenty-five micrograms of the exosome protein samples was dried with a SpeedVac (Thermo Fisher Scientific) and resuspended in iTRAQ dissolution buffer (0.5 mol/L triethylammonium bicarbonate (TEAB), pH 8.5). The protein samples were reduced at 60 °C for 30 min using the iTRAQ reduction buffer (tris-2-carboxyethyl phosphine, TCEP), then alkylated in the dark with iodoacetamide at 37 °C for 30 min. The protein samples were dried using a SpeedVac after being digested with sequencing grade modified trypsin (V511A, Promega, Madison, WI, USA). The peptides were subsequently reconstituted in 10 μL of iTRAQ dissolution buffer and mixed with 30 μL of iTRAQ labelling reagents (Applied Biosystems Inc, Foster City, CA, USA) overnight at 37 °C. The iTRAQ-labelled samples were pooled and desalted with Sep-Pak C18 cartridges (Waters, Milford, MA, USA). The mixtures were dried and re-suspended in 0.5% trifluoroacetic acid before being loaded onto a C18 column (EASY-Spray™, Thermo Fisher Scientific) and separated using a 0.1% formic acid solution with different acetonitrile concentrations (5–80%). The sample was analyzed using a Q ExactiveTMHF mass spectrometer (Thermo Fisher), and the raw MS data were queried using the Mascot search algorithm (version 2.5, Matrix Science, London, UK) against the Swiss-Prot human protein database via Proteome Discoverer (version 2.1, Thermo Fisher Scientific) software.

### 4.4. Expression of the Candidate Protein

Enzyme-linked immunosorbent assay kits were used to assess the protein levels of VWF (EHVWF, Invitrogen, Waltham, MA, USA), CAMP (CSB-EL004476HU, CUSABIO, Houston, TX, USA), PSMB2 (CSB-E17836h, CUSABIO), and Apolipoprotein C-II (APOC2: EHAPOC2, Invitrogen) in the serum and exosomes.

### 4.5. Cell Culture

The HCC cancer cell lines, Huh-7 cells and HepG2 cells, were cultured in Dulbecco’s modified Eagle’s medium (DMEM) (11965092, GIBCO^TM^, Waltham, MA, USA) supplemented with 100 units/mL of penicillin and streptomycin (15140122, GIBCO^TM^) and 10% fetal bovine serum (16000044, GIBCO^TM^) at 37 °C in a humidified 95% air/5% CO_2_ atmosphere. The cells were sub-cultured twice a week at a split ratio of 1:5. The Huh-7 cells were used for the proliferation assay and measurement of the cell cycle distribution. The HepG2 cells were used for measuring the protein expression using Western blotting.

### 4.6. Antibodies and Recombinant Protein

The primary antibodies and recombinant human CAMP protein utilized in this study were commercially available and included the following: monoclonal rabbit anti-GAPDH (ab181602, Abcam, Cambridge, UK), polyclonal rabbit anti-Cathelicidin (OSC00009W, Invitrogen), and recombinant human LL37/Cathelicidin Protein (LS-G2242, LSBio., Lynnwood, WA, USA). The secondary antibodies used for Western blotting included horse radish peroxidase (HRP)-conjugated goat anti-rabbit IgG antibodies purchased from GE Healthcare.

### 4.7. WST-1 Proliferation Assay

The CytoScan™ WST-1 Cell Cytotoxicity Assay (11644807001, Roche, Basel, Switzerland) was used to measure the Huh-7 cell proliferation. Briefly, 2 × 10^4^ Huh-7 cells were seeded in 96-well plates per well with 100 μL of culture media overnight. The serum treatment involved adding 20% serum from healthy or HCC patients to a well and culturing for 48 h. Subsequently, 10 μL of WST-1 assay dye solution was added to each well and incubated at 37 °C for 4 h. Finally, the plates were measured at 450 nm using a microplate reader (TECAN, Lake Zürich, Switzerland). The CAMP functional assay used 20% HCC serum supplemented with 1000 ng/mL of CAMP protein. Antibody neutralization used 20% healthy serum supplemented with CAMP antibody (1:1000) and 20% HCC serum was supplemented with IgG antibody (1:1000). The CAMP protein treatment supplemented 100 μL of culture media with a series of concentrations of CAMP (0 to 1000 ng/mL). Subsequently, the plates were measured at 450 nm after 24, 48, and 72 h.

### 4.8. Distribution of Cell Cycles Measured by Flow Cytometry

The cell cycle analysis involved staining the Huh-7 cells with a propidium iodide staining solution (51-66211E, BD Pharmingen). Briefly, the Huh-7 cells were treated with 1000 ng/mL of CAMP protein, harvested, and fixed with 70% ethanol after 48 h. Subsequently, the cells were washed with PBS and resuspended in binding buffer at a concentration of 1 × 10^6^ cells/mL. The cells were stained with propidium iodide at room temperature for 15 min in the dark. An LSR II flow cytometer (BD Biosciences, Franklin Lakes, NJ, USA) was used to examine the cell distribution throughout the cell cycle, and the results were analyzed using FlowJo^®^ software window version 10.8.

### 4.9. Statistical Analysis

All the statistical analyses were performed using the GraphPad Prism 5 (version 5.01). Mean ± standard error (SEM) was presented as the variables. Pairwise comparisons were performed using the Mann–Whitney test and were represented with a *p* value. All the statistical tests were two-tailed, and differences were considered to be significant at *p* < 0.05.

## 5. Conclusions

We presented evidence indicating a decrease in the CAMP protein levels in the serum of patients with HCC. A reduction in CAMP protein correlates with an increase in HCC cell proliferation. Conversely, elevated CAMP protein levels are associated with reduced cell proliferation and a significant delay in the G1-S transition. These findings suggest that CAMP may function as a tumor suppressor in the context of HCC.

## Figures and Tables

**Table 1:** Differential expression of exosome serum proteins in patients with hepatocellular carcinoma (HCC) compared to healthy controls.

Protein ID	*p*-Value	Fold-Change
HIST1H4A	0.002	2.743
APOC2	0.008	2.513
MMP9	0.009	−1.636
IGFALS	0.013	−1.612
IGLL5	0.014	−1.636
SHBG	0.015	−1.628
IGFBP3	0.022	−1.508
CAMP	0.028	1.711
PSMB2	0.034	1.842
VWF	0.043	1.538
LTF	0.044	−1.546

## Data Availability

Not applicable.

## Supplementary Materials

The following supporting information can be downloaded at: https://www.mdpi.com/article/10.3390/ijms242115652/s1.
