# Supplementary figures and images for: Cathelicidin Antimicrobial Peptide Acts as a Tumor Suppressor in Hepatocellular Carcinoma
Source: Int J Mol Sci. 2023 Oct 27;24(21):15652. doi: 10.3390/ijms242115652 (PMC10647698; doi:10.3390/ijms242115652)

Supplemental Figure S1.

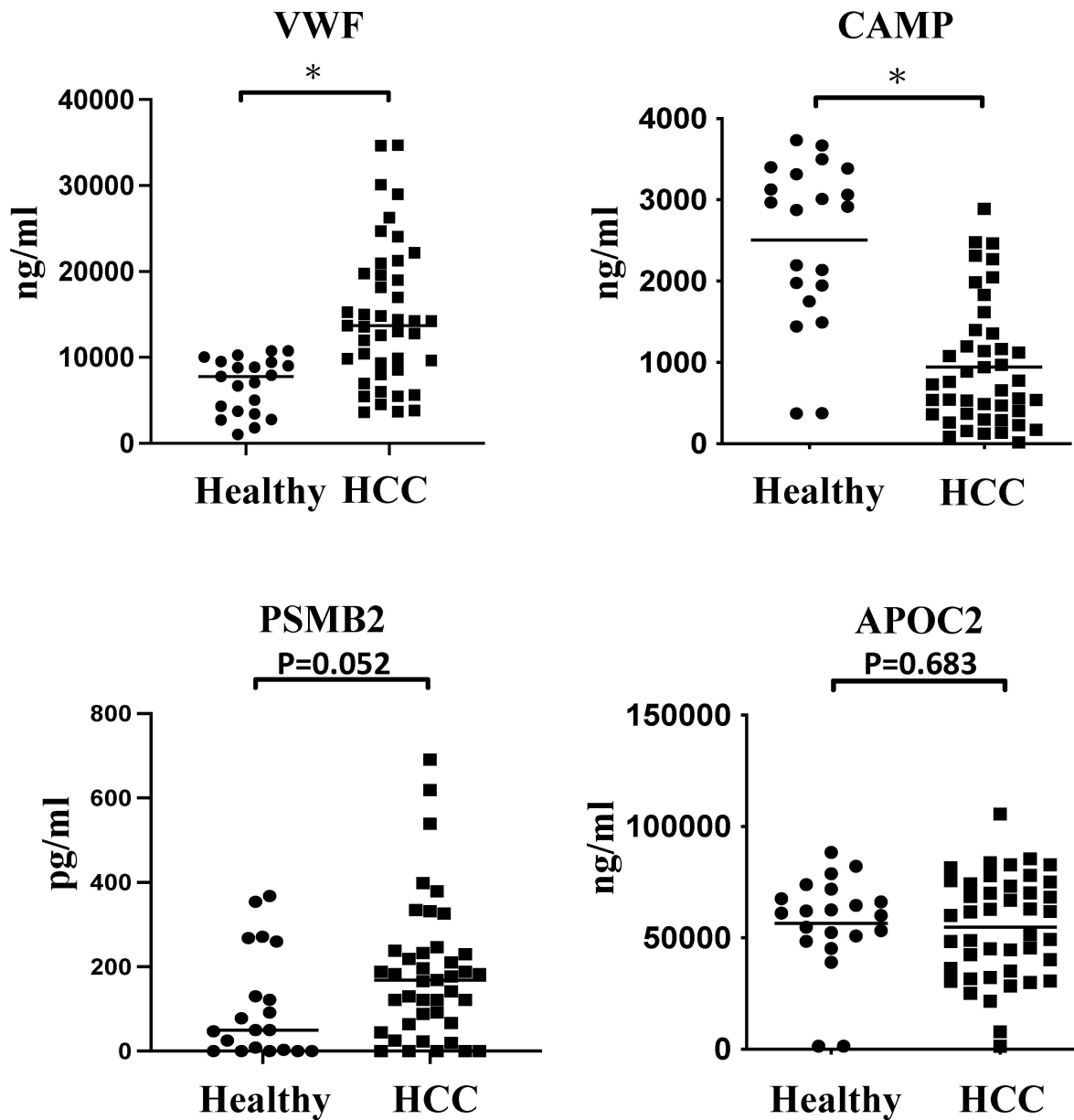

Figure S1: The expression of candidate protein in serum. \*  $p < 0.05$ .

Supplement: Supplementary file 1 [file ijms-24-15652-s001.zip › Supplemental Figure S1.pdf]

Supplemental Figure S2.

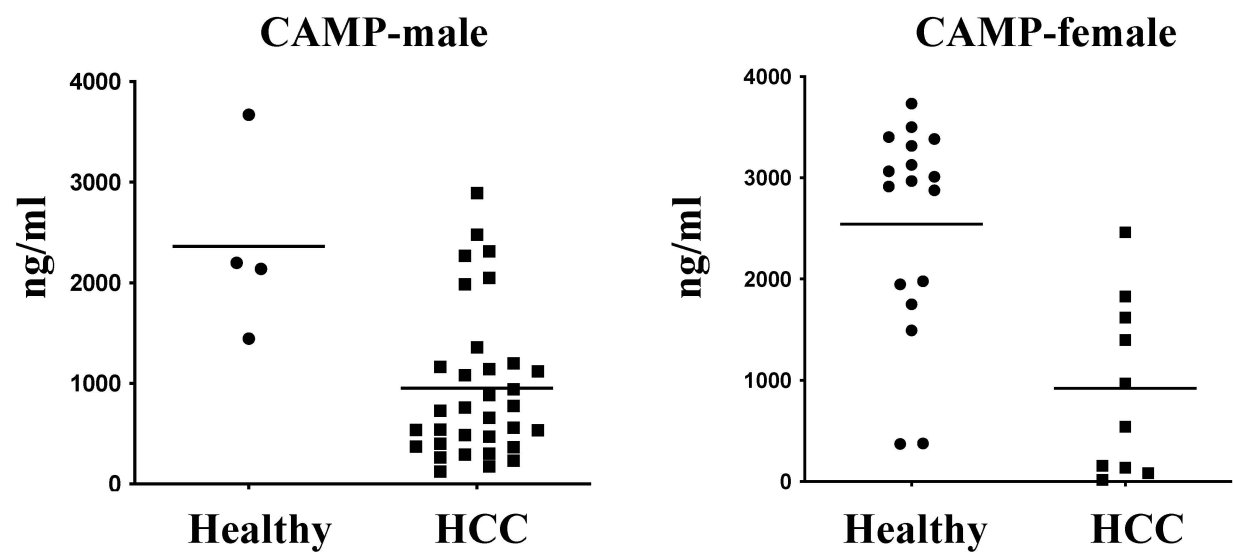

Figure S2: The expression of CAMP protein in male and female.

Supplement: Supplementary file 1 [file ijms-24-15652-s001.zip › Supplemental Figure S2.pdf]
